# Editorial: Madness and creativity—yes, no or maybe?

**DOI:** 10.3389/fpsyg.2015.01055

**Published:** 2015-07-22

**Authors:** Anna Abraham

**Affiliations:** Department of Psychology, School of Social, Psychological and Communication Sciences, Leeds Beckett UniversityLeeds, UK

**Keywords:** psychopathology, creative thinking, divergent thinking, mental illness, psychosis, psychosis proneness, creative cognition

There is something inherently appealing about the idea that creativity and psychopathology are inextricably linked. The eagerness with which this idea is perpetuated and often exaggerated was evidenced most recently in the media frenzy following the publication of a genome-wide association study which demonstrated what in effect was a modest genetic association between creativity and psychosis (Power et al., 2015).

For most investigators of the madness-creativity nexus, the question is not really answered with the categorical and binary choice of whether or not there is an association. Advocates of the “No” camp answer in the negative because they are unconvinced by the quantity and/or quality of evidence provided to support the connection. The same evidence is gauged by “Yes” advocates as constituting enough proof for the claim. It is only by venturing below the surface to understand the actual nature of the association that one can see why this is such a divisive issue. A variety of questions emerge. What is the strength and shape of this association? Is the relation mechanistic? Does one emerge as an epiphenomenon of the other? Is the similarity merely superficial? Is this a question worth asking? Are we asking the wrong question?

The purpose of this Research Topic was to motivate theorists and researchers in the field to take a stance in answering this question given the available evidence thus far (Kaufman, 2014). It is very telling that none of the 14 contributions advocated a resounding “Yes” verdict. The reason for this is straightforward. It is patently clear that the evidence to make a strong claim in the affirmative (all highly creative people have some form of mental illness; all people who have some form of mental illness are highly creative) simply does not exist. So any arguments of deductive reasoning that follow from either of these false premises would be invalid.

The “No” camp has one flag-bearer who, on the basis of grounds such as paucity of empirical evidence, selective data reporting, heterogeneity in types of mental illness, and heuristics-based reasoning behind the link, asserts not only that there is no positive relationship between creativity and mental illness, but that the relationship is in fact negative (Dietrich, 2014). From this standpoint, it is good mental health that leads to more creativity as the need to be creative is part of the self-actualization drive that sits atop the hierarchy of needs pyramid (Maslow, 1943).

In not taking a clear side on the debate, the “Maybe” (or “Yes, but”) camp provides a rich variety of perspectives that seek to uncover the dynamics of the relation between creativity and psychopathology. Some provide methods-based grounds for why the association can be both positive and negative. One commentary addresses the issue of sampling which, as the cross-sectional distribution of creative productivity is highly skewed, gives rise to divergent findings depending on which part of the distribution is being sampled (Simonton, 2014). Another focuses on the metric of information processing biases which are held to orchestrate the connection between creativity and psychopathology (Abraham, 2014). As this relationship follows an inverted-U as opposed to a linear function, it can result in evidence for associations in either direction. A case in point on how evidence of the creativity-psychopathology link is necessarily tied to the type of creativity measure being employed as well as to various forms of psychopathology is showcased in one of the original research articles (Zabelina et al., 2014). That a coherent picture can only be drawn with the explicit consideration and unambiguous acknowledgment of the nature of the construct under study, in terms of definition, operationalization, measures of assessment and populations sampled to assess the association, was highlighted in one of the opinion articles (Fisher, 2015).

Drawing from evolutionary mechanisms that are held to underlie the core components of creativity: novelty (through generators of variation) and usefulness (through generators of fit selection), one postulation is that psychopathology may stem from the extreme ends of these operating principles—psychosis in the case of novelty and autism in the case of usefulness (Jung, 2014). The need to distinguish between different types of psychopathology in relation to creativity, especially in light of the potentially contradictory findings that often result, is captured effectively in one of the original research articles, where creative performance was positively correlated with the analytical/systemizing facets of autistic spectrum characteristics and negatively correlated with the social/empathizing elements of the same (Takeuchi et al., 2014). Others have emphasized that any resemblance in the performance of highly creative people with certain forms of psychopathology is limited to novelty generation as, unlike in the case of psychopathology, highly creative individuals exert efficient control in evaluating the appropriateness of their ideational output (Fink et al., 2014).

Some perspectives showcase brain-based approaches in verifying the link between creativity and psychopathology. Relatively global differences in terms of brain organization, such as via hemispheric asymmetry, are among the earliest ideas that have been put forward to characterize the association (Lindell, 2014). The alternative approach is to focus on specific brain regions and networks. Given the predominant role played by the prefrontal cortex in orchestrating virtually all facets of higher-order function, one means of assessing mechanisms of creative cognition is in terms of prefrontal function and dysfunction. The evidence paradoxically indicates that both enhanced and diminished creative function can result from damage to different parts of this brain structure when evaluating spontaneous versus controlled aspects of the creative process (de Souza et al., 2014). One network-based hypothesis holds that the creativity-psychopathology link is an epiphenomenon that results when the neurocognitive tradeoff between rule-based/top-down systems (prefrontal) and data-driven/bottom-up systems (sensorimotor) is compromised (Ramey and Chrysikou, 2014). This vulnerability often leads to an increase in output quantity (fluency), which in turn gives rise to an increased likelihood of output quality (novelty/uniqueness). An alternate conceptualization of balance between two regulatory systems as mediating the creativity-psychopathology link is that of stability versus flexibility in neural network dynamics, specifically in relation to dopamine and response entropy (Bilder and Knudsen, 2014).

Clinically-based perspectives turn the tide of this dialogue on its head by exploring the alternate possibility that undergoing psychopathological states is what motivates afflicted individuals to seek creative avenues in order to improve their psychological health and well-being (Forgeard and Elstein, 2014). A vital insight of this perspective is that the drive may not be to increase creative output *per se* but to enhance crucial competencies such as flexibility and self-efficacy, which are related but not analogous to creativity. Other accounts focus on the need to consider that the presence of specific personality traits which often accompany psychopathological states, such as openness to experience, may serve as protective factors by channeling the chaotic drive for novelty generation in a productive manner (Kaufman and Paul, 2014).

In bringing these different perspectives together in one common forum, the hope is that this collective effort at addressing this intriguing question will lead to further constructive dialogue and debate in the scientific arena by adding more substance and rigor to discussions of the association between creativity and psychopathology.

## Conflict of interest statement

The author declares that the research was conducted in the absence of any commercial or financial relationships that could be construed as a potential conflict of interest.
